# Isopathy: A Fatal Error

**Published:** 1881-11

**Authors:** Ad. Lippe

**Affiliations:** Philadelphia


### ISOPATHY: A FATAL ERROR.

AD. LIPPE, M. D., PHILADELPHIA.

It is a fatal error to claim isopathy to be Homœopathy. There is no lack of *departures* from the teachings of our Healing-Art, no end to the fatal errors committed by men who profess to belong to the exclusive school of medicine called by its founder, Homœopathy. Hardly have we exposed one fatal error in showing that Homœopathy is not to be confounded with eclecticism; hardly have we shown the folly of "The Historian," who proclaimed (on page 801 of the Transactions of the World's Convention, Historical volume,) that a defunct eclectic school should go on record as a homœopathic institution; hardly have we shown that such a declaration must be construed into an acknowledgment that Homœopathy and eclecticism were synonymous, when arose the President of the World's Homœopathic (?) Convention, held in London, 1881, and boldly, there and then, declared in his address "*that we* (the Congress and its members) *do not, by so acting, pledge ourselves to any exclusiveness in practice.*"* If language means anything this declaration is to the effect *that we* are eclectics to all intents and purposes. We were much gratified to find that these bold bolters were honest enough to make this public confession. If there is anything in the strict inductive method of Hahnemann, these eclectics will by force of logical sequences find themselves compelled to cease "trading in a name," and, not being acceptable to the Old School, set up for themselves as pure eclectics.

Now there comes a new departure. Unproved *but* highly diluted nosodes with new laws, supplementary to the sole universal therapeutic law of the similars, are paraded before the homœopathic school. It was hoped that a paper by Dr. P. P. Wells, published in this journal for August, on "Unproved Remedies," would be sufficient to put at rest this new departure, but if we so believed, we were in error. We are confronted by a staunch defender of this new departure, by a strong homœopathist, who attempts to defend isopathy, trying to make it appear as a part of our therapeutics; who claims further, that cases of cures with highly potentized unproved nosodes should, by all means, be published in a strictly homœopathic journal, and seems to imply that it is right and proper for the International Hahnemannian Association to accept and defend these isopathists. That ignorant men should confound homœopathy with eclecticism is to be deplored, and the ignorant who do not feel able to accept Hahnemann's teachings, who never professed to accept them fully, who never pledged themselves to any exclusiveness in practice, must be left to enjoy the darkness they love so much. But when men who have pledged themselves to an exclusive practice suddenly turn around and revive an almost forgotten departure, Lux's isopathy, and when they claim a recognition of this revived departure, we feel it to be our duty to expose this new fatal error in all its hideousness.

Lux was the father of isopathy, and based his healing method on the principle "*Æqualia æqualibus curantur.*" The modern isopathists claim it to be a law of cure that the products of a disease taken from one individual, when highly potentized, will cure the same disease in other individuals. Under this newly revived *law*, *Tuberculinum* will cure *tuberculosis*, *Cariesin* will cure *caries*,

---

*"Our only covert peculiarity is that we ally ourselves to institutions known as 'homœopathic.'"—President's Address.

*Syphilinum* will cure *syphilis*. They also claim that highly potentized cucumber will cure the ill effects from eating cucumbers and eradicate any long-standing idiosyncrasy. In proof of these claims we are offered facts in the shape of related cures with unproved but highly potentized isopathic remedies, and are asked "what will you do with these facts?" Why, accept them of course for what they are worth, but we do not accept the deductions these isopathists would draw from these facts, remembering well the accepted axiom that "Facts alone prove nothing." All these facts prove is that these isopathic remedies have an effect on the human organism.

Such was the situation when Cullen, in his Materia Medica, dwelt on Cinchona and the reported cures of intermittent fever by this drug. But when he accepted the facts as he found them, i. e., that Cinchona had cured some cases of intermittent fever and failed to cure other cases, he did not claim it to be a specific, but very sensibly asked the question then unsolved, under what circumstances it would cure cases of intermittent fever? Hahnemann solved the question by proving, first on himself and later on others, the sick-making properties of the drug. Our isopathists are now just in the same position Cullen found himself at the end of the last century; they find that products of diseases have medicinal properties, and that is all. Among all the best known and best proved products of a disease stands first and foremost *Psorinum*. Would it not be preposterous to claim that *Psorinum* could cure all cases of the itch? Has it cured any such cases? And what will become of the law, *æqualia æqualibus curantur* if *Psorinum* has failed to cure all, or many, or any cases of the itch? *Psorinum* was proved, and will forever remain an important curative agent, when properly applied under the law of the similars; so may probably all other products of disease become valuable curative agents after exhaustive provings have been made. If isopathy, as it is now attempted to be foisted on Homœopathy, were a true method of healing the sick it would be necessary to show that it possessed universal applicability. What would an isopathist do for hooping cough, or hysteria, or the great host of nervous diseases? What then, if "*Æqualia æqualibus curantur*" is not of universal applicability? What then, if "*Similia similibus curantur*" has been found to be of universal applicability for the cure of the sick? The one, a failure, can surely not be foisted on the other which has fully been tried and is a success. And now for an illustration to show that the *deductive* method adopted by the isopathists is a fallacy, and that the only reliable method is the strictly *inductive* method of Hahnemann. The friends of newly vived isopathy relate a case in which a gentleman, never able to eat cucumbers, and induced to partake of this vegetable, was at once siezed with the usual violent pains he so often had experienced after eating them; a dose of highly potentized cucumber was administered to him and his pain ceased, and we are furthermore assured that ever since then (May, 1876) he has been able to eat cucumbers with impunity. Now the deductive method would argue: (1st) Whosoever cannot eat cucumbers with impunity will be cured of this idiosyncrasy by a single dose of highly potentized cucumber; (2d) This may apply to all other ailments arising from eating certain kinds of food; if one feels ailments of any kind after eating potatoes let him take a dose of highly potentized potato, and under our newly discovered law, proved to be correct by the case above stated, he must recover speedily. So much for the deductive method, which surely cannot be successful if applied as the isopathists propose. Now we find a symptom under the provings of *Alumina* (Chn. Diseases by Hahnemann, symptom 424), "after eating potatoes, pain in the stomach, nausea, feels inclined to vomit and then colic in the abdomen." The isopathists, and men who do not accept the strictly inductive method of Hahnemann, would declare *Alumina* to be a specific for persons not able to partake of potatoes with impunity—make it a key-note to be sure—but the men who accept Hahnemann's inductive method claim that *Alumina* will cure just such pains and discomforts as are described in symptom 424, and if other discomforts arise after eating potatoes they look for another remedy. What will the isopathists, standing by their deductive method, do with symptom 424? What is *that symptom* to them? What are all of Hahnemann's rules and regulations to them? Have they not discovered a *new* law? If a highly potentized potato, according to their newly discovered law, must cure all ailments from eating potatoes, of what possible use can symptom 424, or any part of our materia medica be to them? Till we find this new departure to obviate the tedious study of our materia medica, till the isophathists show that their newly discovered laws work well in *all* cases of disease, we, of the old guard, shall hold on to the old landmarks. Here is another case good for the isopathists to reflect on. A gentleman about thirty-two years old, for many years resorting only to the strictest homœopathic treatment when sick, presented himself, suffering from a newly acquired gonorrhœa. Taking in consideration the totality of the patient's symptoms, regardless of pathological notions as to an inflammatory first stage of the disease, regardless of the newly re-discovered isopathic laws, the patient received one dose of *Sulphur* 21 M (F. C.). Seven days after this dose had been taken the patient reported improvement and seven days later he was found to be "cured," and remained so. Were we to follow the deductive method adopted by the isopathists, who are in the habit of deducing laws based on a single fact, we would proclaim *Sulphur* (when highly potentized) a specific for gonorrhœa; with just as much recklessness as they proclaimed *Medorrhin* a specific for gonorrhœa, because that remedy has benefited (never cured) a case of gonorrhœa. What does the above related fact prove? Why, that a diseased condition can now as well as in Hahnemann's days be cured mildly, safely and permanently if we only follow the Master's injunctions faithfully. *Sulphur* will cure only such cases of disease as the symptoms, as recorded under its sickmaking properties, when administered to *healthy* individuals, show a similarity to, and never otherwise. Do the isopathists claim that *Medorrhin* is a specific for gonorrhœa? If they are consistent believers in their newly discovered labor-saving mode of cure, they have to put it that way, for, if they themselves show any doubts about the general and universal applicability of their newly discovered *laws*, they expose themselves to well-merited ridicule.

The attempt to foist eclecticism on Homœopathy has been repelled and the unfortunate actors in this farce have been shown a praiseworthy precedent, set by *one* of their former organs, the New York *Homœopathic Times*, which journal has acted honestly by dropping its *homœopathic* title. There can be no doubt about the course these men, who now find themselves so cruelly exposed as having "traded under a false name," will be forced to take, and as Homœopathy has beens haking off the eclectics, so will it shake off the isopathists, who now attempt to fasten themselves on the homœopathic school. We have *gently hinted* at the great difference which exists between Homœopathy and isopathy. It is to be hoped that, in future, the advocates of this revived heresy will not ask for a recognition outside of their own organization; as they belong to no known system of medicine, as they claim to have discovered new *laws* of cure, it would be well for them to hoist their own flag and proclaim themselves opposed to Homœopathy, allopathy and eclecticism, and unite under their own banner on which is written their motto—*Æqualia æqualibus curantur.*

